# The AktiWeb study: feasibility of a web-based exercise program delivered by a patient organisation to patients with hip and/or knee osteoarthritis

**DOI:** 10.1186/s40814-022-01110-3

**Published:** 2022-07-20

**Authors:** Kenth Louis Joseph, Hanne Dagfinrud, Kåre Birger Hagen, Kristine Røren Nordén, Camilla Fongen, Ole-Martin Wold, Rana S. Hinman, Rachel K. Nelligan, Kim L. Bennell, Anne Therese Tveter

**Affiliations:** 1 Center for treatment of Rheumatic and Musculoskeletal Diseases (REMEDY), Diakonhjemmet Hospital, Oslo, Norway; 2 Faculty of Medicine, Institute of Health and Society, University of Oslo, Oslo, Norway; 3 Division of Health Service, Norwegian Institute of Public health, Oslo, Norway; 4 Norwegian League against Rheumatism, Oslo, Norway; 5 Centre for Health, Exercise and Sports Medicine, Department of Physiotherapy, School of Health Sciences, Faculty of Medicine Dentistry & Health Sciences, The University of Melbourne, Melbourne, Australia

**Keywords:** Osteoarthritis, Follow-up strategy, Patient organisations, Web-based exercise program, Physical activity

## Abstract

**Background:**

Patient organisations may be an under-utilised resource in follow-up of patients requiring long-term exercise as part of their disease management. The purpose of this study was to explore the feasibility of a web-based exercise program delivered by a patient organisation to patients with hip and/or knee osteoarthritis (OA).

**Methods:**

In this pre–post feasibility study, patients aged 40–80 years with hip and/or knee OA were recruited from Diakonhjemmet Hospital. The 12-week intervention was delivered through a patient organisation’s digital platform. Feasibility was evaluated by proportion of eligible patients enrolled, proportion of enrolled patients who provided valid accelerometer data at baseline, and proportion completing the cardiorespiratory exercise test according to protocol at baseline and completed follow-up assessments. Patient acceptability was evaluated for website usability, satisfaction with the initial exercise level and comprehensibility of the exercise program. Change in clinical outcomes were assessed for physical activity, cardiorespiratory fitness and patient-reported variables.

**Results:**

In total, 49 eligible patients were identified and 35 were enrolled. Thirty (86%) of these attended baseline assessments and provided valid accelerometer data and 18 (51%) completed the maximal cardiorespiratory exercise test according to protocol. Twenty-two (63%) patients completed the follow-up questionnaire, and they rated the website usability as ‘acceptable’ [median 77.5 out of 100 (IQR 56.9, 85.6)], 19 (86%) reported that the initial exercise level was ‘just right’ and 18 (82%) that the exercise program was ‘very easy’ or ’quite easy’ to comprehend. Improvement in both moderate to vigorous physical activity (mean change 16.4 min/day; 95% CI 6.9 to 25.9) and cardiorespiratory fitness, VO_2peak_ (mean change 1.83 ml/kg/min; 95% CI 0.29 to 3.36) were found in a subgroup of 8 patients completing these tests. Across all patient-reported outcomes 24–52% of the patients had a meaningful improvement (*n* = 22).

**Conclusion:**

A web-based exercise program delivered by a patient organisation was found to be feasible and acceptable in patients with hip and/or knee OA.

**Trial registration:**

ClinicalTrials.gov, NCT04084834 (registered 10 September 2019). The Regional Committee for Medical and Health Research Ethics south-east, 2018/2198. URL: Prosjekt #632074 - Aktiv med web-basert støtte. - Cristin (registered 7 June 2019).

**Supplementary Information:**

The online version contains supplementary material available at 10.1186/s40814-022-01110-3.

## Key messages

- The treatment needs of the large group of patients with chronic diseases such as osteoarthritis impose a significant burden on the healthcare system, and patient organisations may be a valuable resource with untapped potential in follow-up of patients requiring long-term exercise as part of their disease management.
- Although some adjustments are needed, a web-based exercise program focusing on cardiorespiratory fitness and delivered through a patient organisation seem feasible, acceptable and safe for patients with hip and/or knee osteoarthritis.
- To provide evidence on the effectiveness of the program, a randomised controlled trial should be conducted.

## Introduction

Exercise is a well-documented treatment option for most chronic diseases [1–3], and in line with this, physical activity (PA) and exercise is recommended as first-line treatment for patients with hip and knee osteoarthritis (OA) [4, 5]. It is well known that many patients with hip- and knee OA are less physically active than recommended [6–8], and our recent study showed that at the age of 40, people with OA already had a significantly shorter walking distance on the 6-min walking test compared to an age-matched reference group [9]. Due to increasing life-expectancy in the general population, the prevalence of OA is expected to rise in the next decades [10, 11]. To limit functional decline and development of co-morbidities, this large patient group should be encouraged to include regular exercise as part of their disease management.

Different types of exercise programs (i.e. strengthening and/or aerobic) show similar benefits regarding OA-related symptoms [12], while aerobic exercise also has a particular potential to prevent serious cardiovascular comorbidities which is highly prevalent in OA populations [3, 13]. For beneficial health outcomes, long-term exercise is needed, but adhering to a prescribed exercise program over time is challenging without support [14]. As OA is highly prevalent [15] the treatment needs of patients with OA impose a significant burden on healthcare systems [11, 16]. The development of innovative, scalable and effective treatments and follow-up strategies is urgently required.

Peer-support is recognised as an effective way to strengthen patients’ self-efficacy and motivation to support long-term adherence to exercise [13, 14, 17], and patient organisations may be an under-utilised resource in support and follow-up of patients who need long-term exercise as part of their treatment plan. Patient organisations can provide resources such as web-based platforms for interaction and distribution of information, as well as contact with experienced peer-supporters. Web-based delivery of self-management programs, including exercise, is shown to be an effective method for improving pain and physical functioning in patients with musculoskeletal conditions, including OA [18, 19]. Thus, by providing specially adapted exercise programs along with support from a network of experienced and educated peers, patient organisations may fulfil the role of a valuable collaborator and an extended resource for the healthcare service.

In this project, a web-based, peer-supported aerobic exercise program for patients with hip- and/or knee OA (the AktiWeb study) was developed in close cooperation between a patient organisation and physiotherapists and a sport scientist at Diakonhjemmet Hospital. The program was developed as a stepwise, progressive model, in which the exercise dose was individually adjusted based on patients self-reported data in a web-based diary. In order to facilitate further studies on effectiveness and implementation of this model [20], the aim of this study was to explore the feasibility of a web-based exercise program delivered by a patient organisation to patients with hip and/or knee OA.

## Methods

### Design

This study was a pre-post single-arm feasibility study. The study was evaluated and approved by the Regional Committee for Medical and Health Research Ethics (REK south-east, 2018/2198) and the Data Protection Officer at Diakonhjemmet Hospital (reg. no. 00138). The study was pre-registered in ClinicalTrials.gov (NCT04084834) and recruitment started in October 2019. Reporting of the study follows the Consolidated Standards for Reporting Trials (CONSORT) 2010 statement: extension to randomised pilot and feasibility trials [21] and the template for intervention description and replication (TIDieR) checklist and guide [22] as appropriate.

### Patient recruitment and data collection

**Fig. 1:**
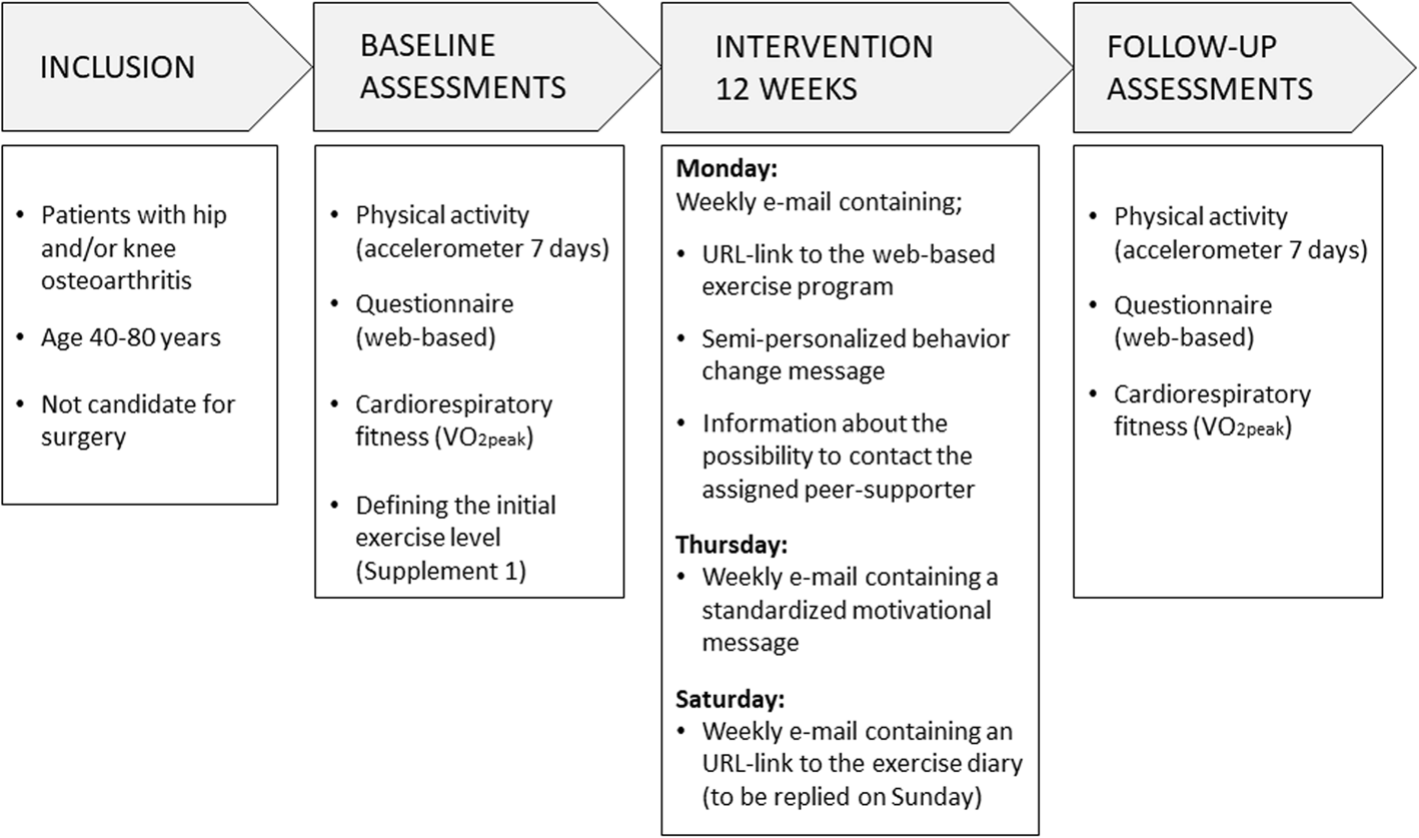
The main elements of the AktiWeb fesibility study

Participants were recruited among patients referred to Diakonhjemmet Hospital for surgical consultation due to radiographic hip and/or knee OA. Among patients consulting the surgeon, 49 were pre-screened and identified for possible inclusion. A project associate (CF, AC, KLJ) gave verbal and written information about the study and conducted a more thorough screening against eligibility and exclusion criteria. Inclusion criteria were patients with hip and/or knee OA aged 40–80 years and not considered a candidate for surgery. Exclusion criteria were patients unable to understand or write Norwegian, unable to walk unaided and continuously for 15 min, had relatives with sudden death before 40 years of age, or first-degree relatives with hypertrophic cardiomyopathy or other heart disease, had absolute or relative contradictions to maximal exercise testing (established coronary heart disease and/or symptoms of other heart disease, indication of heart disease during PA, previously confirmed abnormal electrocardiogram measures, systolic blood pressure > 200 mmHg or diastolic blood pressure > 115 mmHg, acute systemic infection with fever, bodily pain or swollen lymph nodes, chronic infection) [23]. Those agreeing to participate provided written consent. Reasons for unwillingness to participate were recorded. Following consent, patients received an URL-link directed to a web-based questionnaire (in Service for Sensitive Data, TSD, University of Oslo, Norway) and an accelerometer to wear for 7 consecutive days before their scheduled baseline assessment at the hospital (within 1–3 weeks). After the baseline assessment, patients initiated the 12-week AktiWeb intervention and were scheduled for a follow-up assessment after 12 weeks. The main study elements are illustrated in Fig. 1.

### Sample size

Guidelines for sample size calculation in feasibility studies is not established [21], but it is important that the sample size is large enough to provide sufficient information for running a future randomised controlled trial. In previous studies, sample size in feasibility and pilot studies has been reported to be between 30 and 36 patients [24], while Sim and Lewis [25] have suggested a sample size of at least 50 patients. Due to the multiple components that needed to be tested in the current study (e.g. peer-support, the AktiWeb website, exercise program and exercise diary), we aimed to enrol 50 patients to ensure sufficient data to evaluate feasibility.

### The AktiWeb intervention

The AktiWeb intervention was developed in close collaboration between Diakonhjemmet Hospital and a patient organisation (Norwegian League against Rheumatism, NRF). A patient research partner was involved in all phases of the project. The development are according to the newly published UK Medical Research Councils’ framework for developing complex interventions [26]. The intervention comprised five components:

#### Peer-support

Two experienced and educated peer-supporters from NRF’s network of peers took part in the study. An NRF peer-supporter is a voluntary person with a rheumatic disease who has been educated as a peer-supporter by NRF. The peers’ main resource is considered to be the competence to provide knowledge, experience, inspiration, guidance and support related to living with a chronic disease. The patients were reminded weekly via e-mail about the possibility to contact the assigned peer-supporter if needed (the peer-supporters name and mobile number were included). The peers recorded number of contacts and time used per contact.

#### The AktiWeb website

The website was designed on NRF’s official website especially for study participants and contained seven main sections with brief information about recommended core treatment, exercise and symptoms, benefits of exercise, adaption and adjustment of exercise, endurance exercise, PA and the exercise programs. The OA specific information was based on the non-pharmacological treatment recommendations for management of hip- and knee OA [4, 27, 28] and PA recommendations for people with OA [29]. An URL-link to the website was included in the weekly e-mail sent to the patients on Mondays.

#### The AktiWeb exercise program

The program focused on aerobic exercise and general PA and comprised five different levels with three exercise sessions per week: one interval session, one pyramid interval session and one low intensity session (shown in detail in Additional file 1). Each session was described and graphically illustrated with suggestions on how and where to exercise and included the BORG Rating of Perceived Exertion (RPE) scale to describe intensity [30]. The initial exercise level was defined based on baseline assessments according to predefined criteria including VO_2peak_, PA habits, pain during activity and experience with interval exercise (shown in detail in Additional file 1), while the exercise level in the following weeks was adjusted by the project manager (ATT) according to responses in the digital diary. If an exercise diary reply was missing the patients received the same exercise program as the previous week. An URL-link to each weekly exercise program were included in the e-mail sent on Mondays. To ensure acceptability and uptake of the exercise program, input from the patient research partner influenced that the initial level was somewhat lower than recommended [29], but with an aim to increase to recommended level during the intervention period.

#### The exercise diary

Each Saturday, patients received an URL-link to the exercise diary by e-mail in which they were asked to report (on Sunday) the number of exercise sessions performed and if these were completed according to the prescribed program. Patients who completed ≤ 2 of the prescribed sessions were asked to report barriers for not complying with the exercise program. These barriers (forgot, too tired, joint hurts so I cannot exercise, worried exercise is causing pain/injury, exercise is not helping, boring, lack of time, life stress, none of the alternatives apply to me) were adapted from a theory-informed behaviour change message program [31] designed to overcome major barriers to exercise adherence in people with OA [14]. If a reply was missing on Monday morning, the patients received an e-mail reminder.

#### Motivational behaviour change messages

The patients received unique motivational behaviour change messages twice a week by e-mail; one was standardised to motivate for exercise (i.e. ‘*Sticking to your exercise program has benefits beyond just your OA’*), while the other was semi-personalized based on exercise diary response and was designed to overcome reported barriers or to reinforce continued exercise adherence (i.e. message related to lack of time: ‘*Think about what time of day you are less tired. Make a plan to do your exercises at that time of day. Commit to it each week*’). The messages were selected by a project associate (ATT) from a library (198 messages incorporating 20 behaviour change techniques to overcome common barriers to exercise and to facilitate exercise participation) developed by researchers at the University of Melbourne (Australia) according to the Behaviour Change Wheel Framework [31]. The messages were translated into Norwegian and some adjustments were made to fit the aim of the project and to account for seasonal variations in Norway. All weekly e-mails were sent by the project manager (ATT).

### Feasibility

#### Logistics

Feasibility of the logistics was evaluated by calculating proportion of eligible patients enrolled and the proportion of enrolled patients providing valid accelerometer data at baseline, completing the indirect maximal cardiorespiratory exercise test according to protocol at baseline, returning exercise diaries (as well as number of received diaries) and providing follow-up data. The logistics of intervention delivery were evaluated by time used on delivering the intervention (exercise programs and motivational messages) and time used by peer-supporters (calculated as minutes per patient/week).

#### Patient acceptability

Patient acceptability of interventional components was evaluated at follow-up by asking the patients about usability of the website, satisfaction with the initial exercise level according to predefined criteria, comprehensibility of the exercise program and the degree to which different components motivated them to adhere to the exercise program.

#### Clinical outcomes

To inform future studies about relevant clinical outcomes, change in PA, cardiorespiratory fitness and patient-reported outcomes from baseline to follow-up was reported. For patient-reported outcomes, also proportions of patients with meaningful change were reported.

#### Measures

Website usability was evaluated by using the System Usability Scale (SUS) comprising ten standardised questions, scored on a 5-point Likert scale, which was calculated into a sum score ranging from 0 (low usability) to 100 (high usability) [32] where scores above 70 are considered acceptable usability [33]. Satisfaction with the initial exercise level according to predefined criteria was evaluated by asking patients if the initial exercise level was suitable (‘too easy’, ‘just right’ or ‘too hard’). Comprehensibility of the exercise program was evaluated by asking if the exercise program was easy to comprehend (5-point Likert scale ranging from ‘very difficult’ to ‘very easy’). The degree to which the different study components motivated the patients to adhere to the exercise program was assessed for seven study components, each scored on an 11-point numeric rating scale (NRS, 0 = was not motivating at all, 10 = was very motivating): performing a treadmill test prior to the exercise program, consulting a physiotherapist prior to the intervention, the tailored exercise program, receiving weekly exercise programs, weekly reporting in the exercise diary, receiving weekly motivational messages and performing a treadmill re-test after 12 weeks.

PA was assessed by accelerometers (ActiGraph GT3X+, Pensacola, FL) prior to baseline assessment and after follow-up assessment at the hospital. Patients were asked to wear the accelerometer on their right hip, using an adjustable elastic belt, during waking hours (except for water-based activities) for seven consecutive days. Data were downloaded and processed (ActiLife Software v6.13.3, ActiGraph, LLC) from the vertical axis in 60-s epochs, and we applied the Troiano algorithm to aggregate data on wear-time, counts per minute (CPM), and moderate to vigorous PA (MVPA, > 2019 CPM) [34].

Cardiorespiratory fitness (VO_2peak_) was assessed on a treadmill (Woodway PPS55) according to a modified Balke protocol [35]. Age-predicted maximal heart rate [211– (0.64*age)] [36] was estimated, and heart rate was monitored (Polar FT1; Polar, Kempele, Finland) to supervise physiological exertion during the test. Patients rated their perceived exertion using the BORG RPE scale [30]. VO_2peak_ (ml/kg x min) was estimated based on incline and speed at the test end stage in combination with age and weight, using previously developed equations [37]. A submaximal single-stage protocol [38] was prepared for patients unable or unwilling to perform a maximal exercise test. The submaximal test results were excluded from analyses on cardiorespiratory fitness.

Joint-related disability was measured using the Hip disability and Osteoarthritis Outcome Score (HOOS) and Knee injury and Osteoarthritis Outcome score (KOOS) (www.koos.nu). Normalised scores ranging from 0 (extreme disability) to 100 (no disability) were calculated according to scoring instructions (www.koos.nu), and a change of 10 points was considered a meaningful change [39–41].

Numeric rating scales (NRS, 0–10) were used to measure pain (0 = no pain; 10 = worst imaginable pain), disease activity (0 = no symptoms; 10 = very bad), and fatigue (0 = no fatigue; 10 = worst imaginable fatigue) during the last week. A 30% relative change is considered a clinically important change in NRS pain [42, 43], and this was applied to all the NRS scales to define a meaningful change.

The utility index of the EQ-5D-5L (– 0.59 to 1, 1 = perfect health) was used to assess health-related quality of life (www.euroqol.org), using a value set derived from England [44]. Health status was measured with the EQ-5D visual analogue scale (VAS, 0–100, 0 = worst imaginable health; 100 = best imaginable health). For the EQ-5D utility index, a ≥ 0.07-point improvement and a ≥ 0.05 worsening was defined as meaningful changes and for the EQ-5D VAS, a ≥ 10 point change was defined as a meaningful change [45].

The Norwegian Arthritis Self-Efficacy Scale (ASES) was used to assess perceived arthritis specific self-efficacy measured by a pain subscale (5 items) and a symptoms subscale (6 items), each scored on a 5-point Likert scale (1–5) ranging from ‘very certain’ to ‘very uncertain’, in which the sum score of each subscale were converted to a 0–100 scale (100 = high self-efficacy) [46].It is recommended to use ASES to assess self-efficacy following patient education programs for people with rheumatic diseases, but responsiveness of the ASES is reported to be poor with standard response means of 0.13–0.19 (< 6%) [47]. Conservatively, we applied a difference of ≥ 10% as an indication of meaningful change.

The Exercise Beliefs and Exercise habits was used to assess exercise self-efficacy measured by four subscales with each item scored on a 5-point Likert scale (1-5) ranging from ‘strongly disagree’ to ‘strongly agree’: exercise self-efficacy (4 items, 4–20, 20 = best score), barriers to exercise (3 items, 3–15, 15 = best score), benefits of exercise (5 items, 5–25, 25 = best score), and impact of exercise on arthritis (8 items, 8–40, 40 = best score) [48]. To aid interpretation of the outcomes in relation to the ASES outcomes, we applied a difference of ≥ 10% to indicate a meaningful change.

#### Participant characteristics

Demographic and clinical characteristics were self-reported and included age, gender, body mass index [kg/m^2^], living arrangements [living alone/living with someone], education level [≥ 1 year of college/university (primary school, upper secondary school) and /< 1 year of college/university (college/university < 4 years, college/university ≥ 4 years)], smoking [yes/no], work status [working full time, not working full time (working part time, sick leave full time, sick leave part time, retired, well-fare, work assessment allowance, staying at home, student)], most troublesome joint [right/left, hip/knee], number of troublesome joints [range 1–9, right/left, hip/knee/ankle/hand or fingers], pain [NRS 0–10, 0 = no pain], disease activity [NRS 0–10, 0 = no disease activity] and number of co-morbid conditions [range 0-15, categorised into 0, 1 and ≥ 2 co-morbid condition(s)].

#### Registration of adverse events

Patients were asked to contact the project coordinator if any adverse event occurred due to the intervention. Adverse events were also recorded by questionnaire at 12-week follow-up and was defined as any adverse event experienced in the last 12 weeks that the patient believed was a result of physical exercise.

#### Statistical analyses

Data are reported as mean and standard deviation (SD), median and interquartile range (IQR, 25th and 75th percentile) or frequencies and percentage. The change in outcome measures was analysed using paired sample t-test, given as mean change (95% confidence interval), and the proportions of patients with meaningful change and non-meaningful change are shown in percentages. IBM SPSS Statistics version 25 was used for statistical analyses.

## Results

**Fig. 2:**
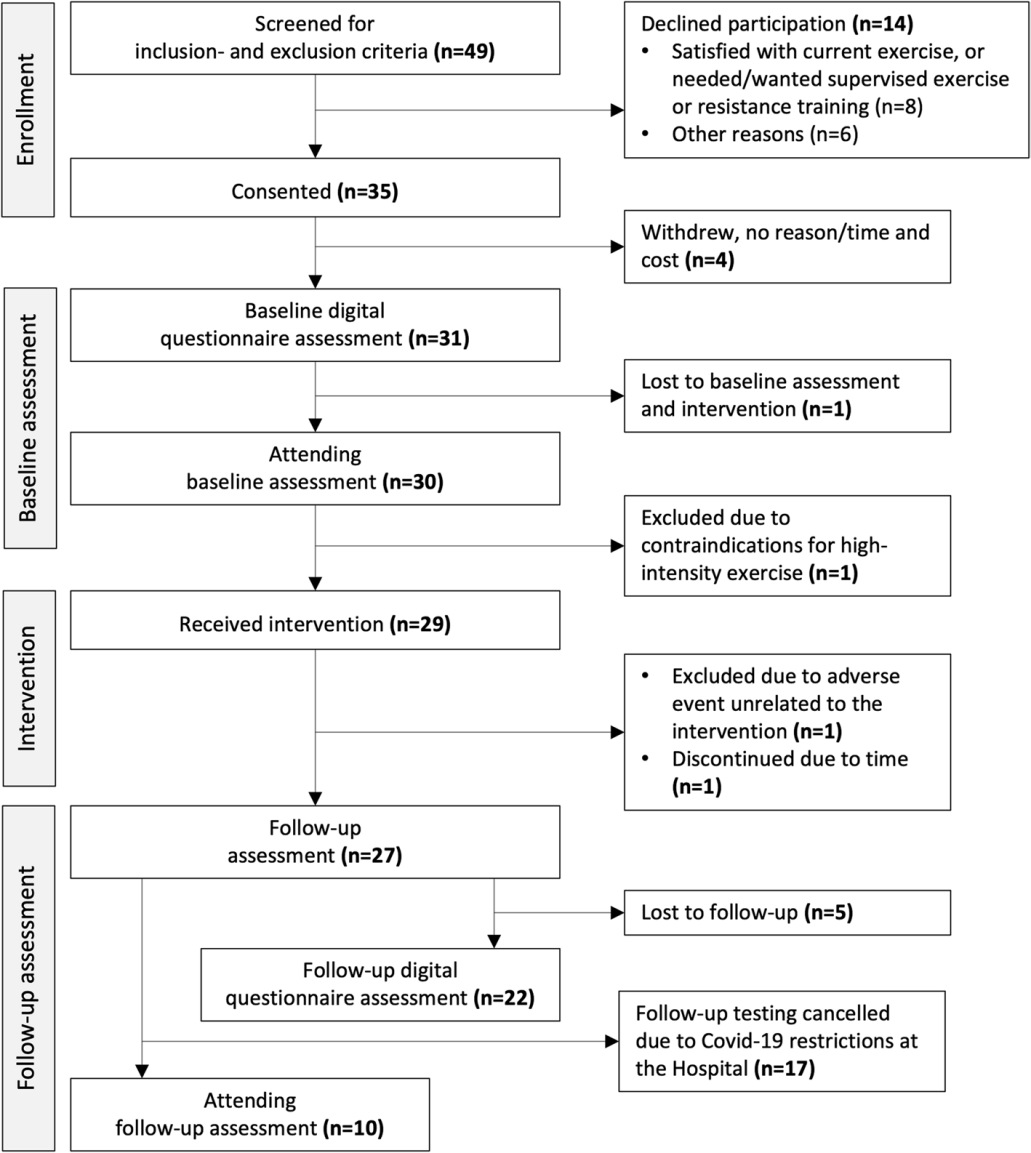
Flowchart of patient recruitment and data assessments in the AktiWeb study

Recruitment to the project began in October 2019 and was terminated in March 2020 due to the COVID-19 pandemic and enrolment thus ceased with 35 participants (Fig. 2). Demographics of the enrolled patients with baseline data are shown in Table 1.

**Table 1:** Characteristics of patients with hip and/or knee osteoarthritis who attended baseline assessments (*n* = 30)

Demographics	
Age, years, mean (SD)	63.3 (9.5)
Female, *n* (%)	21 (70.0)
Body mass index, kg/m^2^, mean (SD)	30.4 (6.7)
Living arrangement, living alone, *n* (%) ^a^	11 (38)
Education level, ≥ 1 year of college/university, *n* (%)	19 (63)
Non-smokers, *n* (%)	29 (97)
Working full time, *n* (%)	13 (43)
Clinical characteristics	
Pain (NRS, 0–10, 0 = no pain), median (IQR)	5.0 (3.0, 6.3)
Disease activity (NRS, 0–10, 0 = no disease activity), median (IQR)	5.0 (3.8, 7.0)
Most troublesome joint, *n* (%)	
Knee (right or left)	26 (87)
Hip (right or left)	4 (13)
Total number of troublesome joints (range 0–9), *n* (%)	
1 to 4 joints	25 (83)
5–9 joints	5 (17)
Number of co-morbid conditions (range 0–15), *n* (%)^b^	
No co-morbid conditions	10 (33)
One co-morbid condition	14 (47)
2 to 4 co-morbid conditions	6 (20)

^a^*n* = 29 due to missing data

^b^Data based on the question: Is your health currently affected by one or more of these medical problems (each answered by yes/no): high blood pressure, angina/infarction/other cardiac disease, asthma/bronchitis/other pulmonary disease, allergy/rhinitis/eczema/, sciatica, cerebral haemorrhage/cerebral stroke, cancer disease, neurological disease (in brain- or nerve tissue), diabetes, metabolic disease, mental/psychological disease, kidney disease, liver disease, ulcer or other stomach disease, anaemia or other blood disease

### Logistics

We identified 49 eligible patients and 35 were enrolled. Among these, 86% (30/35) attended baseline assessments. At baseline compliance with wearing the accelerometer was mean (SD) 6.1 (1.0) valid days with mean (SD) 13.8 (1.3) hours per day. Twenty-nine patients performed a submaximal (*n* = 9) or maximal (*n* = 20) cardiorespiratory exercise test. The peer-supporters were not contacted by the patients. Logistic outcomes are shown in Table 2.

**Table 2:** Logistics of the AktiWeb study in patients with hip and/or knee osteoarthritis

Logistics	Outcome
Enrolled	
Proportion of eligible patients enrolled	71% (35/49)
Assessment	
Proportion of patients providing valid accelerometer data at baseline assessment	86% (30/35)
Proportion of patients completing maximal cardiorespiratory exercise test according to protocol at baseline assessment	51% (18/35)
Proportion of patients returning exercise diary	77% (27/35)
Received exercise diaries per patient (0–12), median (range)	11 (1–12)
Proportion of enrolled patients providing data at 12-week follow-up assessments	63% (22/35)
Intervention delivery	
Time resources used on delivery of exercise programs and motivational messages, minutes per week/patient, mean (SD)	7.3 (1.1)
Time resources used by peer-supporters, minutes per week/patient	0^a^

^a^The peer-supporters were not contacted

### Patient acceptability

**Fig. 3:**
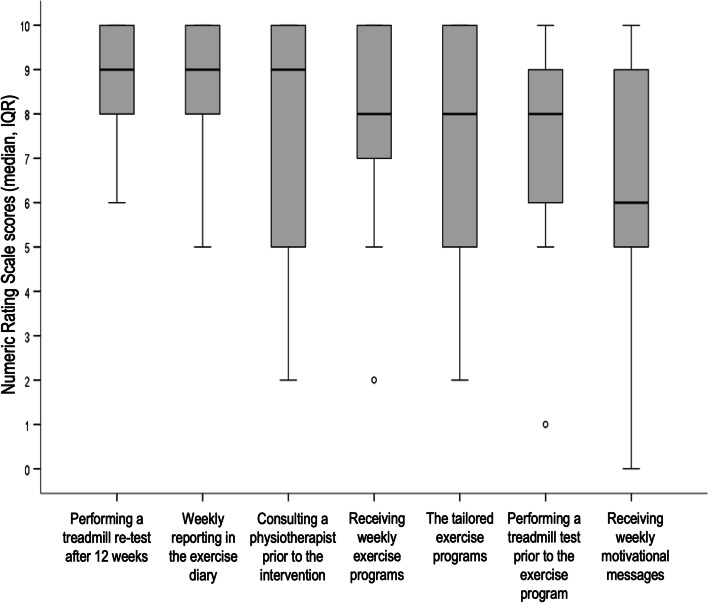
The degree to which study components motivated patients to adhere to the exercise program (*n* = 22)

The website usability was rated as ‘acceptable’ with a median (IQR) SUS rating of 77.5 (56.9, 85.6), *n* = 22. Patient satisfaction with the initial exercise level according to predefined criteria was reported to be ‘just right’ by 19 (86%) patients, ‘too easy’ by two (9%) patients and ‘too hard’ by one (5%) patient. The exercise program was found to be ‘very easy’ to comprehend by 13 (59%) patients, ‘quite easy’ by five (23%), ‘uncertain’ by three (14%) and ‘very difficult’ by one (5%). The degree to which the different study components motivated the patients to adhere to the exercise program are shown in Fig. 3.

### Clinical outcomes

In a subset of patients, both PA and cardiorespiratory fitness (VO_2peak_) increased from baseline to follow-up, and across all patient-reported outcomes 24–52% of the patients reported change in scores that could be categorised as a meaningful improvement (Table 3).

**Table 3:** Outcome measures and proportions of patients with meaningful change or no change

	*N*	Baseline Mean (SD)	Follow-up Mean (SD)	Mean change (95% CI)	Proportion improved	Proportion no change	Proportion worsened
Physical activity							
Counts per minute/day	8	295.2 (70.7)	390.2 (110.8)	94.9 (45.0 to 144.8)			
MVPA minutes/day	8	33.2 (17.1)	49.6 (22.2)	16.4 (6.9 to 25.9)			
Cardiorespiratory fitness, VO_2peak_ (ml/kg/min)	8	25.05 (5.93)	26.88 (6.79)	1.83 (0.29 to 3.36)			
HOOS/KOOS, normalised scores (0–100, 100 = best score)							
Symptoms	21	46.0 (17.0)	55.3 (17.1)	9.3 (4.6 to 14.0)	48%	48%	5%
Pain	20	55.1 (19.5)	61.5 (20.2)	6.4 (1.5 to 11.3)	40%	55%	5%
ADL	21	62.7 (18.7)	71.8 (19.2)	9.1 (5.3 to 13.0)	52%	43%	5%
Sports/Rec	20	35.3 (26.1)	40.3 (29.1)	5.0 (− 2.4 to 12.3)	30%	45%	25%
QoL	21	34.7 (13.6)	42.7 (17.8)	8.0 (1.8 to 14.3)	43%	43%	14%
Numeric Rating Scales (NRS), 0–10, 0 = no pain							
NRS pain, last week	20	5.2 (2.2)	4.5 (2.4)	0.7 (− 0.1 to 1.4)	30%	60%	10%
NRS fatigue, last week	22	3.8 (3.1)	3.1 (2.7)	0.6 (− 0.5 to 1.8)	45%	41%	14%
NRS disease activity, last week	22	5.4 (2.1)	4.5 (2.1)	0.9 (− 0.1 to 1.9)	41%	41%	18%
Health-related quality of life							
EQ-5D-5L utility score (− 0.59 to 1)	16	0.79 (0.14)	0.85 (0.11)	0.06 (0.03 to 0.09)	38%	56%	6%
EQ-5D VAS (0–100, 100 = best health)	17	61.9 (15.1)	70.5 (18.3)	8.6 (1.2 to 16.0)	47%	41%	12%
Arthritis Self-Efficacy Scale							
Pain, mean (0–100)	20	57.4 (13.6)	56.5 (12.2)	0.9 (− 7.4 to 9.1)	30%	50%	20%
Symptoms, mean (0–100)	21	54.6 (10.9)	58.1 (-14.6)	− 3.5 (− 9.0 to 2.0)	38%	48%	14%
Exercise beliefs							
Self-efficacy, sum score (4–20)	21	14.8 (2.4)	16.8 (2.3)	− 2.0 (− 3.5 to − 0.4)	48%	43%	10%
Barriers to exercise, sum score (3–15)	20	11.7 (2.1)	11.8 (2.1)	− 0.1 (− 0.9 to 0.7)	25%	55%	20%
Benefits of exercise, sum score (5–25)	21	20.0 (3.2)	20.7 (2.5)	− 0.8 (− 0.2 to 0.5)	30%	55%	15%
Impact of exercise on arthritis, sum score (8–40)	21	31.9 (4.6)	33.2 (4.5)	− 1.3 (− 2.7 to 0.1)	24%	76%	0%

*SD* standard deviation, *95% CI* 95% confidence interval, *MVPA* moderate to vigorous physical activity, *VO2peak* peak oxygen uptake, *HOOS* Hip disability and Osteoarthritis Outcome Score, *KOOS* Knee injury and Osteoarthritis Outcome score, *ADL* function in daily living, *Sport/Rec* function in sport and recreation, *QoL* hip/knee-related quality of life

### Adverse events

Three patients reported minor events due to transitory pain (back, knee, and unknown site), while one patient reported a moderate adverse event involving consultation with a general practitioner due to chest pain, after which the patient completed the intervention.

## Discussion

The main objective of this feasibility study was to examine the logistics and patient acceptability of a 12-week web-based exercise program for patients with hip and/or knee OA. The delivery and follow-up of the program was overall found to be feasible and acceptable, and a subset of the participants showed improved PA level and cardiorespiratory fitness after completion of the program. Only a few minor adverse events were reported, thus, the intervention is regarded as safe for patients with hip and knee OA. The promising results of this feasibility study can be used for planning a methodologically sound and robust randomised controlled trial.

Innovative follow-up strategies that facilitate patients with chronic conditions to self-manage are needed to support the future healthcare system and patient organisations may be an under-utilised resource in the support and follow-up of patients with OA. Among the resources that patient organisations can offer are web-based platforms for interaction and delivery of disease management programs, as well as access to experienced peer-supporters. In this study, a stepwise, progressive exercise program was developed in close collaboration with a patient organisation, and the program was delivered on their website. This approach showed promising results, indicating that patient organisations can be an alternative pathway of disease management and follow-up for patients with chronic conditions.

The exercise program was delivered on a website and the interaction with the participants was based on e-mail which is a feasible method for delivering interventions to large numbers of people with OA. A future development could be to provide a mobile application for more efficient and automated delivery of intervention components. The use of the e-mail system to deliver a weekly website-link to the exercise program and provide an individually tailored behaviour change message was time consuming for the project associate. Digitally automated exercise programs and messages [31] could be utilised in the future for even more efficient delivery.

Social support and peer encouragement are known to be important factors for exercise adherence [13, 14, 17]. However, the assigned peer-supporters were not utilised by patients in the present study. A possible reason may be that the behaviour change messages may have reduced the need for additional support during the 12-week program as similar messages have previously been shown to support adherence to home-base exercise in knee OA [49]. Qualitative research could establish the reasons why peer-supporters were not contacted by patients, and whether peer-support could be provided based on the patients’ needs.

The motivational messages used in this study were developed specifically for patients with hip or knee OA, based on behaviour change techniques linked to barriers and facilitators of exercise adherence in this patient group [14, 31]. The messages, delivered via SMS, have been evaluated in a clinical trial in 110 people with knee OA, showing that adherence to a resistance exercise program was higher in the group that received the messages by SMS compared to the control group who did not receive messages [49]. Combined with a web-based self-directed exercise program, the SMS messages have also been shown to improve pain and function at 24 weeks in people with knee OA [50]. In the current study, most participants reported that the messages to some degree motivated them to adhere to exercise, but other interventional components (i.e. receiving weekly exercise programs and reporting in an exercise diary) were rated as even more important motivational factors. Collectively, these results show that methods for motivation and follow-up are appreciated by people with OA and should be used to enhance patients’ adherence to exercise.

PA and exercise are important core treatments to maintain or improve functional capacity and cardiorespiratory fitness [3, 5]. In this study, a subset of the participants improved PA and cardiorespiratory fitness (VO_2peak_) equal to the results reported in a recent meta-analysis including studies on patients with knee OA who followed aerobic exercise [51]. Even if the number of participants in our study was limited due to the COVID-19 pandemic, the positive results were supported also in the self-reported measures of pain and function. Thus, it seems that patients with lower limb OA can follow aerobic exercise programs outside of healthcare settings, and obtain improvement in physical fitness. However, with the uncontrolled nature of our feasibility study we cannot conclusively attribute changes in clinical outcomes to our intervention. Future robust randomised controlled trials are needed to definitively determine treatment efficacy.

Objective, valid testing of physical capacity is needed when providing individually tailored exercise programs to patients and is valuable to inform goal setting and to monitor adherence to prescribed exercise [3, 23]. In this study, accelerometery was used to evaluate patients’ level of PA and a treadmill exercise test was used to measure fitness in a subgroup of the patients, and both were considered acceptable. Although all patients who attended baseline assessments provided valid baseline data for PA in our study, others have reported 10-28% missing at follow-up among individuals with OA (i.e. due to non-valid wear time or technical issues with the accelerometer) [52, 53]. Additionally, 31% of the participants had to perform a submaximal exercise test for assessment of fitness level. Thus, for use in large patient groups, simpler methods such as non-exercise-based fitness calculators [3] or easily conducted performance-based measures (i.e. 6-min walk test) [54] could be used to achieve the purpose of testing.

In this study, the exercise dosage was individually adjusted by a project associate based on the weekly digital exercise diaries in which adherence to prescribed exercise was reported. Self-reporting adherence to exercise may function as self-monitoring, which is recognised as an important facilitator for exercise adherence [14, 29]. As a further development to enhance the advantages of self-monitoring, the data from exercise diaries could be combined with data on self-reported OA symptoms [55]. Graphical illustrations could be produced to visualise the association between exercise and disease burden, which would be a useful tool for patients in optimising their dosage of PA.

The main limitation of this study is that restrictions due to the COVID-19 pandemic stopped the inclusion and limited the follow-up assessment of patients. Further, before recruitment the participants were pre-screened and selected from a cohort of patients referred to specialised healthcare for surgical consultation, and the results may therefore not be generalisable to the total OA population. Another limitation is that we did not predefine criteria to determine whether to stop or proceed with a future larger trial. However, we have made a thorough discussion of results to inform a possible future RCT. Even if strict exclusion criteria for high intensity exercise testing was applied, only one patient was excluded due to this. Thus, following the ACSM guidelines [23] for high intensity testing should be done in future trials.

## Conclusion

A web-based exercise program with a stepwise, progressive design delivered by a patient organisation was found to be feasible, acceptable and safe in patients with hip and knee OA, and positive results were found for PA, cardiorespiratory fitness and several patient-reported outcomes. The findings in this feasibility study can inform future trials as our promising results support that patient organisations can play the role as a valuable resource in long-term follow-up of patients with chronic conditions, and thereby potentially alleviate the healthcare system.

## Supplementary Information

**Additional file 1.** The exercise program.

## Data Availability

The datasets generated and analysed during the current study are not publicly available because the data include identifiable data on individuals. De-identified data may be made available from the corresponding author on reasonable request.

## Abbreviations

BORG RPE: BORG Rating of Perceived Exertion
CPM: Counts per minute
EQ-5D VAS: EQ-5D Visual Analogue Scale
HOOS: Hip disability and Osteoarthritis Outcome Score
KOOS: Knee injury and Osteoarthritis Outcome score
MVPA: Moderate to Vigorous Physical Activity
NRF: Norwegian League against Rheumatism
NRS: Numeric Rating Scale
OA: Osteoarthritis
PA: Physical activity
REK: Regional Committee for Medical and Health Research Ethics
SD: Standard deviation
SUS: System usability scale
VO_2peak_: Peak oxygen uptake
CI: Confidence interval
