# Supplementary material for: The AktiWeb study: feasibility of a web-based exercise program delivered by a patient organisation to patients with hip and/or knee osteoarthritis
Source: Pilot Feasibility Stud. 2022 Jul 20;8:150. doi: 10.1186/s40814-022-01110-3 (PMC9296765; doi:10.1186/s40814-022-01110-3)
Supplement: Supplementary file 1 — Additional file 1. The exercise program. [file 40814_2022_1110_MOESM1_ESM.pdf]

Additional file 1 (the exercise program)

	**Session 1**<br>**Interval exercise**		**Session 2**<br>**Pyramid interval exercise**		**Session 3**<br>**Low intensity PA exercise**		**Criteria for initial (week 1) exercise level**
	F x D<br>(active pause)	BORG RPE<br>(active pause)	F x D<br>(active pause)	BORG RPE<br>(active pause)	F x D	BORG RPE	Clinical evaluation in addition to:
**Level 1**	3 x 3<br>(2-3 min)	13-15<br>(10-12)	5 x 1-3<br>(1-3 min)	13-15<br>(10-12)	1 x 20-30 min	8-12	VO2peak <ref. values; PA habits <1-2/week *or* not performing maximal exercise test.
**Level 2**	4 x 3<br>(2-3 min)	13-15<br>(10-12)	5 x 1-3<br>(1-3 min)	15-17<br>(10-12)	1 x 30-45 min	8-12	VO2peak <ref. values; PA habits ≥1-2/week *or* VO2peak ≥ ref. values; PA habits 1-2/week; NRS pain in activity ≥6.
**Level 3**	4 x 3<br>(2-3 min)	13-15<br>(10-12)	7 x 1-4<br>(1-4 min)	15-17<br>(10-12)	1 x 30-45 min	8-12	VO2peak ≥ref. values; PA habits 1-2/week; NRS pain in activity <6 *or* VO2peak ≥ref. values; PA habits ≥3/week; NRS pain in activity ≥6 *or* VO2peak <10% above ref. values; PA habits ≥3/week; NRS pain in activity <6.
**Level 4**	4 x 4<br>(1-4 min)	15-17<br>(10-12)	7 x 1-4<br>(1-4 min)	15-17<br>(10-12)	1 x 45-60 min	8-12	VO2peak ≥10% above ref. values; PA habits ≥3 per/week; NRS pain in activity <6; performing interval exercise <1-2/week.
**Level 5**	4 x 4<br>(1-4 min)	15-17<br>(10-12)	7 x 1-4<br>(1-2 min)	15-17<br>(10-12)	1 x 45-60 min	8-12	VO2peak ≥10% above ref. values; PA habits ≥3 per/week; NRS pain in activity <6; performing interval exercise ≥1-2/week.

F, frequency (number of intervals); D, duration (minutes per interval); BORG RPE, BORG rating of perceived exertion (intensity); VO_{2peak}, peak oxygen uptake; ref.values, reference values; Physical activity (PA) habits, PA of at least 30 min with increased respiration and heart rate; performing interval exercise, performing regular interval exercise the last three months; NRS, numeric rating scale (0-10).

VO_{2peak} mean reference values (gender and age-category specific) retrieved from: Edvardsen E, Hansen BH, Holme IM, Dyrstad SM, Anderssen SA. Reference values for cardiorespiratory response and fitness on the treadmill in a 20- to 85-year-old population. Chest. 2013;144(1):241-8.
